# Association of inflammatory index with the severity of disease in patients with acute myocarditis: A retrospective observational study

**DOI:** 10.3389/fendo.2025.1597427

**Published:** 2025-08-25

**Authors:** Shipeng Wang, Hanchi Xu, Zhen Guo, Yulin Tian, Xia Guo, Haoxuan Chu, Yushi Wang

**Affiliations:** Department of Cardiovascular Medicine, The First Hospital of Jilin University, Changchun, China

**Keywords:** systemic inflammatory index, systemic immune-inflammation index, acute myocarditis, NPAR, SII, SIRI, AISI

## Abstract

**Purpose:**

This research aimed to investigate the association between neutrophil-percentage-to-albumin ratio (NPAR), systemic immune-inflammation index (SII), systemic inflammation response index (SIRI), and aggregate index of systemic inflammation (AISI) with disease severity in patients diagnosed with acute myocarditis.

**Methods:**

A total of 185 patients were diagnosed with acute myocarditis at the First Hospital of Jilin University between 2018 and 2024. The related values of NPAR, SII, SIRI, and AISI were computed based on the pertinent blood indices that were acquired within 12 hours of admission. The best cut-off values for NPAR, SII, SIRI, and AISI, as well as their associated sensitivity and specificity, were determined using ROC curve analysis in order to assess their predictive usefulness for poor prognosis upon admission.

**Results:**

Patients with fulminant myocarditis exhibited significantly higher NPAR, SII, SIRI, and AISI values compared to those with mild myocarditis. Spearman correlation analysis revealed significant associations between these inflammatory indices and NYHA scores at admission (r = 0.370, 0.296, 0.284, and 0.246, respectively; *P* < 0.01). Multivariate logistic regression analysis identified high NPAR (OR: 5.44 95%, CI:1.81 ~ 16.36, *P=*0.003), SII (OR: 1.01 95%CI:1.01 ~ 1.01, *P=*0.010), SIRI (OR: 1.21, 95%CI:1.06 ~ 1.37, *P=*0.005), and AISI (OR: 1.01 95%CI:1.01 ~ 1.01, *P=*0.007) values as independent risk factors for myocarditis severity.

**Conclusions:**

Our study demonstrated that inflammatory biomarkers - NPAR, SII, SIRI, and AISI - show associations with the severity of acute myocarditis.

## Introduction

Acute myocarditis represents an inflammatory cardiomyopathy marked by immune-mediated myocardial damage triggered by diverse etiologies including viral infections, autoimmune disorders, toxic exposures, and pharmacological agents. Clinical outcomes range from spontaneous resolution to progression toward dilated cardiomyopathy or sudden cardiac death (1–3). Among the adolescent population, it has become the main cause of sudden cardiac death, surpassing the incidence of ischemic heart disease. Current epidemiological data indicate that the incidence of acute myocarditis is 10 to 22 per 100,000 and is on the rise (3, 4). The clinical manifestations of myocarditis are heterogeneous, ranging from mild symptoms such as chest pain and palpitations to life-threatening cardiogenic shock and ventricular arrhythmias. There exists a critical need for validated prognostic biomarkers to facilitate early risk stratification and guide therapeutic interventions in this heterogeneous patient population.

Pathological manifestations of myocarditis include myocyte necrosis, fibrosis, and edema, driven by innate and adaptive immune responses (5). While the exact pathogenic mechanisms remain elusive, contemporary research highlights neutrophils as key mediators of myocardial injury in acute myocarditis (6, 7). Clinical studies demonstrate that elevated neutrophil-to-lymphocyte ratio (NLR) and monocyte-to-lymphocyte ratio (MLR) correlate with prolonged hospitalization in myocarditis patients (8). The neutrophil percentage-to-albumin ratio (NPAR) synergistically combines acute-phase (neutrophils) and chronic-phase (albumin) inflammatory markers, offering a more integrated evaluation of inflammatory status (9). Systemic Immune Inflammation Index (SII) and Systemic Inflammation Response Index (SIRI) as sensitive indicators of inflammatory immune homeostasis, show strong prognostic value in various inflammatory diseases (10). Among acute coronary syndrome patients receiving percutaneous coronary intervention (PCI), both SIRI and aggregate index of systemic inflammation (AISI) independently predict major adverse cardiovascular events (11, 12).

These integrated inflammatory indices provide a clinically feasible and methodologically robust framework for risk stratification. When incorporated with conventional clinical parameters, they enable the development of multidimensional prediction algorithms that optimize both prognostic accuracy and therapeutic decision-making. This study specifically aims to validate the clinical utility of four novel inflammatory indices (NPAR, SII, SIRI, and AISI) for early severity assessment in acute myocarditis patients.

## Materials and methods

### Study population

This retrospective analysis included 185 consecutive adults (aged ≥18 years) diagnosed with acute myocarditis at the First Hospital of Jilin University (January 2018 - December 2024). Exclusion criteria comprised: (1) active malignancy; (2) autoimmune disorders; (3) concurrent systemic inflammation; (4) incomplete clinical documentation.

### Definitions

Acute myocarditis is currently diagnosed by meeting the following criteria: (1) Coronary computed tomography angiography (CTA) or angiography excludes acute coronary syndrome; (2)elevated troponin levels, (3) echocardiographic evidence of ventricular dysfunction without underlying structural heart defects, (4) prodromal illnesses (respiratory or gastrointestinal) within 2 weeks of symptom onset, (5) electrocardiogram changes suggesting acute myocardial injury or arrhythmias, and (6) signs and symptoms of acute heart failure (dyspnea, reduced exercise tolerance, syncope, exertional chest pain, tachypnea, unexplained tachycardia, hepatomegaly, and galloping rhythm).

The presence of significant hemodynamic impairment necessitating the use of positive inotropic medications or ventricular support devices, such as extracorporeal membrane oxygenation (ECMO), left ventricular assist devices, or intra-aortic balloon pumps, is identified as fulminant acute myocarditis (13–15).

### Clinical and laboratory data

Demographic data such as gender, age, vital signs, laboratory results, comorbidities, medication use, management of patients in the Cardiovascular Intensive Care Unit (CICU), electrocardiogram (ECG) and echocardiogram (echo) results, and complications were all routinely evaluated for each participant. Brain natriuretic peptide precursor (BNP) levels, serum troponin, albumin (ALB), C-reactive protein (CRP), neutrophils (N), lymphocytes (L), platelets (PLT), and monocytes (M). The inflammation index and its calculation method are shown in **Table 1**.

**Table 1:** Inflammation index and its calculation method.

Inflammatory index	Calculation formula
NPAR	N/WBC×100/ALB
SII	PLT × [N/L]
SIRI	N × [M/L]
AISI	[N×M×PLT]/L)

### Statistical analysis

We utilized logistic regression analysis to examine the associations between NPAR, SII, SIRI and AISI, and the fulminant myocarditis. Multivariate regression analysis was adjusted for potential confounding factors found to be significant in univariate regression, including age (in years), sex, smoking and drinking status, laboratory data(EF%, cTnI, BNP and CRP) and comorbid conditions (DM, hypertension and history of CVD). Associations between NPAR,SII, SIRI, AISI and myocarditis were evaluated further by checking the diagnostic performance of NPAR, SII, SIRI, AISI for the severity of myocarditis by using the area under the receiver operating characteristics (AUROC). The optimal ROC cut-off point was determined by using the Youden index. All statistical analyses were performed using the Statistical Package for the Social Sciences 25.0 (SPSS; IBM, USA), with a two-tailed *P* < 0.05 considered statistically significant.

## Result

The baseline clinical characteristics of the total cohort (N=183) are detailed in **Table 2**. The majority of myocarditis patients are men, however fulminant myocarditis is more common in women. Compared to the mild myocarditis group, the fulminant myocarditis group had higher age, NYHA scores, Neutrophil, cardiac troponin I (cTnI), CK-MB, BNP, CRP, lactic acid (Lac), aspartate aminotransferase (AST), alanine aminotransferase (ALT), serum creatinine (sCr), NPAR, SII, SIRI and AISI. But lower heart rate, ejection fraction, blood pressure, lymphocyte counts, Monocytes counts (all *P* < 0.05).

**Table 2:** Demographics and clinical characteristics of acute myocarditis patients.

Variables	Total (n = 183)	Fulminant myocarditis (n = 34)	Mild myocarditis (n = 149)	*P*
Age	**30.00 (21.00, 39.00)**	**37.50 (24.25, 46.50)**	**29.00 (20.00, 39.00)**	**0.020**
Gender, n (%)				**<.001**
men	**114 (62.30)**	**10 (29.41)**	**104 (69.80)**	
female	**69 (37.70)**	**24 (70.59)**	**45 (30.20)**	
HR	80.00 (74.00, 92.00)	78.00 (69.25, 111.50)	80.00 (75.00, 90.00)	0.860
SBP (mmHg)	**120.00 (108.50, 125.00)**	**109.00 (90.75, 120.00)**	**120.00 (111.00, 128.00)**	**<.001**
DBP (mmHg)	**75.00 (64.00, 80.00)**	**68.50 (60.00, 75.00)**	**75.00 (65.00, 80.00)**	**0.009**
Smoking, n (%)				**0.008**
Now	**35 (19.13)**	**1 (2.94)**	**34 (22.82)**	
Never/Former	**148 (80.87)**	**33 (97.06)**	**115 (77.18)**	
Drinking, n (%)				1.000
Now	22 (12.02)	4 (11.76)	18 (12.08)	
Never/Former	161 (87.98)	30 (88.24)	131 (87.92)	
Hypertension, n (%)				1.000
Yes	9 (4.92)	2 (5.88)	7 (4.70)	
No	174 (95.08)	32 (94.12)	142 (95.30)	
Diabetes, n (%)				0.453
Yes	3 (1.65)	1 (3.03)	2 (1.34)	
No	179 (98.35)	32 (96.97)	147 (98.66)	
Coronary Heart Disease, n (%)				1.000
Yes	3 (1.64)	0 (0.00)	3 (2.01)	
No	180 (98.36)	34 (100.00)	146 (97.99)	
NYHA, n (%)				**<.001**
I	**54 (29.67)**	**1 (2.94)**	**53 (35.81)**	
II	**72 (39.56)**	**3 (8.82)**	**69 (46.62)**	
III	**23 (12.64)**	**2 (5.88)**	**21 (14.19)**	
IV	**33 (18.13)**	**28 (82.35)**	**5 (3.38)**	
EF%	**59.00 (51.00, 62.00)**	**46.00 (35.00, 55.00)**	**60.00 (56.50, 63.00)**	**<.001**
LVDD (mm)	48.00 (46.00, 50.50)	49.00 (46.00, 52.00)	48.00 (46.00, 50.00)	0.522
Monocytes (10*9/L)	0.59 (0.41, 0.85)	0.58 (0.44, 0.89)	0.60 (0.40, 0.84)	0.564
Neutrophil (10*9/L)	**5.35 (3.95, 7.32)**	**6.95 (5.22, 10.41)**	**5.11 (3.73, 6.86)**	**<.001**
Lymphocytes (10*9/L)	**1.55 (1.09, 2.10)**	**1.33 (0.69, 1.85)**	**1.68 (1.14, 2.15)**	**0.017**
cTnI (ng/ml)	**5.02 (0.68, 12.70)**	**12.40 (9.71, 23.72)**	**2.71 (0.40, 9.09)**	**<.001**
CK-MB (ng/ml)	**17.60 (5.00, 45.00)**	**45.00 (30.60, 60.65)**	**12.45 (2.97, 36.48)**	**<.001**
BNP (pg/ml)	**92.30 (20.23, 452.75)**	**702.00 (250.00, 1020.00)**	**59.30 (16.50, 225.00)**	**<.001**
Hb (g/L)	**140.00 (126.00, 152.00)**	**129.50 (114.25, 141.75)**	**144.00 (128.00, 153.00)**	**<.001**
PLT (10*9/L)	219.00 (176.00, 268.00)	212.00 (169.25, 264.00)	221.00 (178.00, 268.50)	0.424
CRP (mg/L)	22.07 (8.44, 54.21)	29.52 (14.37, 51.84)	19.97 (5.97, 54.32)	0.229
Lac (mmol/L)	**1.30 (0.90, 2.00)**	**2.90 (1.50, 5.60)**	**1.10 (0.83, 1.40)**	**<.001**
HCT	**0.42 (0.38, 0.45)**	**0.38 (0.35, 0.43)**	**0.42 (0.39, 0.45)**	**0.002**
ALB (g/L)	**38.45 (35.10, 41.32)**	**34.90 (31.30, 37.70)**	**39.30 (36.25, 42.00)**	**<.001**
AST (U/L)	**59.35 (31.40, 120.47)**	**192.60 (80.00, 669.70)**	**45.70 (27.55, 89.40)**	**<.001**
ALT (U/L)	**35.05 (22.17, 64.85)**	**77.50 (53.30, 681.60)**	**30.60 (21.15, 55.05)**	**<.001**
SCr (mmol/L)	**64.20 (54.10, 74.70)**	**77.50 (60.10, 115.70)**	**63.70 (53.22, 70.42)**	**<.001**
NPAR	**1.78 (1.50, 2.22)**	**2.26 (1.82, 2.61)**	**1.71 (1.45, 2.07)**	**<.001**
SII	**732.00 (476.17, 1367.71)**	**1263.60 (585.50, 2302.01)**	**688.00 (441.37, 1043.72)**	**0.002**
SIRI	**2.12 (1.01, 4.28)**	**3.41 (2.16, 6.12)**	**1.76 (0.89, 3.62)**	**<.001**
AISI	**433.93 (242.83, 803.03)**	**622.66 (424.41, 1127.78)**	**385.34 (196.80, 774.05)**	**0.003**

Bold values indicate statistical significance.

**Figure 1:**
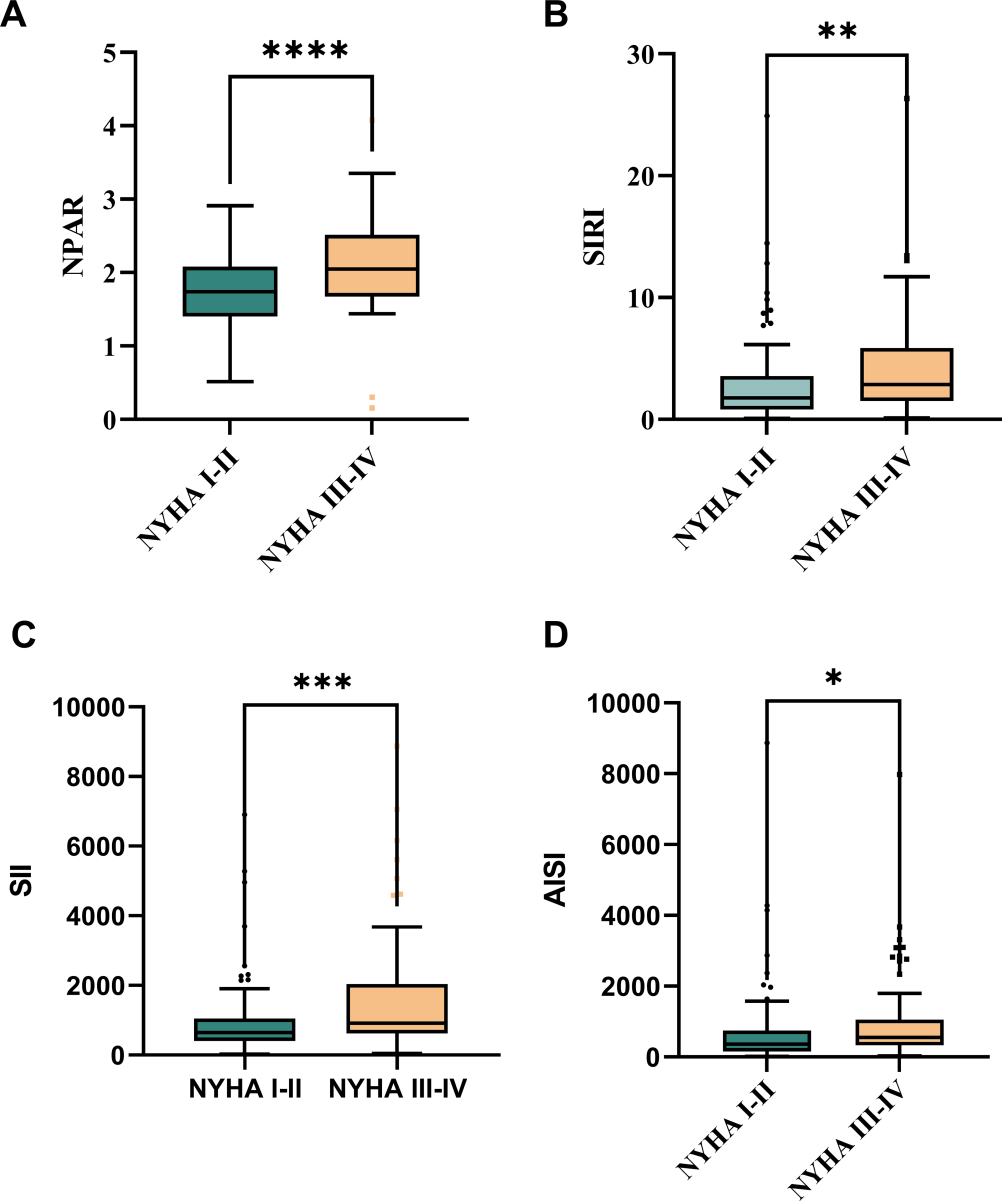
Disparities between admission NYHA scores and NPAR, SII, SIRI, and AISI levels:Compared with patients with NYHA I-II acute myocarditis, NPAR **(A)**, SIRI **(B)**, SII **(C)**, and AISI **(D)** levels in patients with NYHA III-IV acute myocarditis. *:P < 0.05; **:P < 0.01; ***:P < 0.001; ****:P < 0.0001

For patients with myocarditis, Spearman correlation analysis showed significant relationships between the NYHA score at admission and the values of NPAR, SII, SIRI, and AISI (r = 0.370, 0.296, 0.284, and 0.246, *P* < 0.01). Patients with worse cardiac function (NYHA score > III) nonetheless had higher levels of NPAR, SII, SIRI and AISI than patients with comparatively better cardiac function (NYHA score < II) (**Figure 1**).

**Figure 2:**
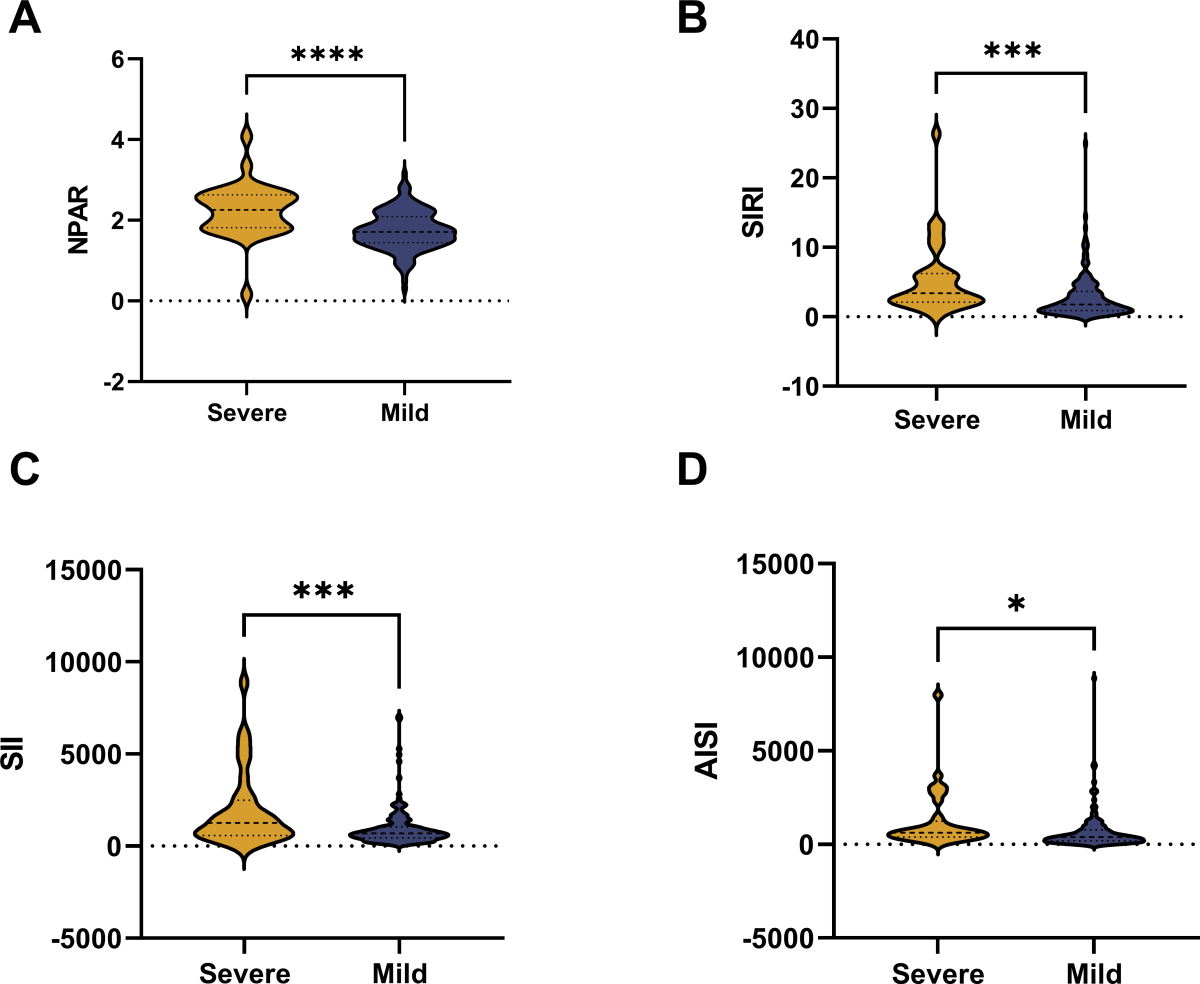
The violin plot of composite inflammatory ratios:Compared with patients with mild acute myocarditis, the levels of NPAR **(A)**, SIRI **(B)**, SII **(C)**, and AISI **(D)** in patients with fulminant acute myocarditis.

The differences in NPAR, SII, SIRI, and AISI between patients with mild acute myocarditis and those with fulminant myocarditis are compared in **Figure 2**. The mild patient group had lower levels of NPAR, SII, SIRI, and AISI than the severe patient group (*P* < 0.05).

**Figure 3:**
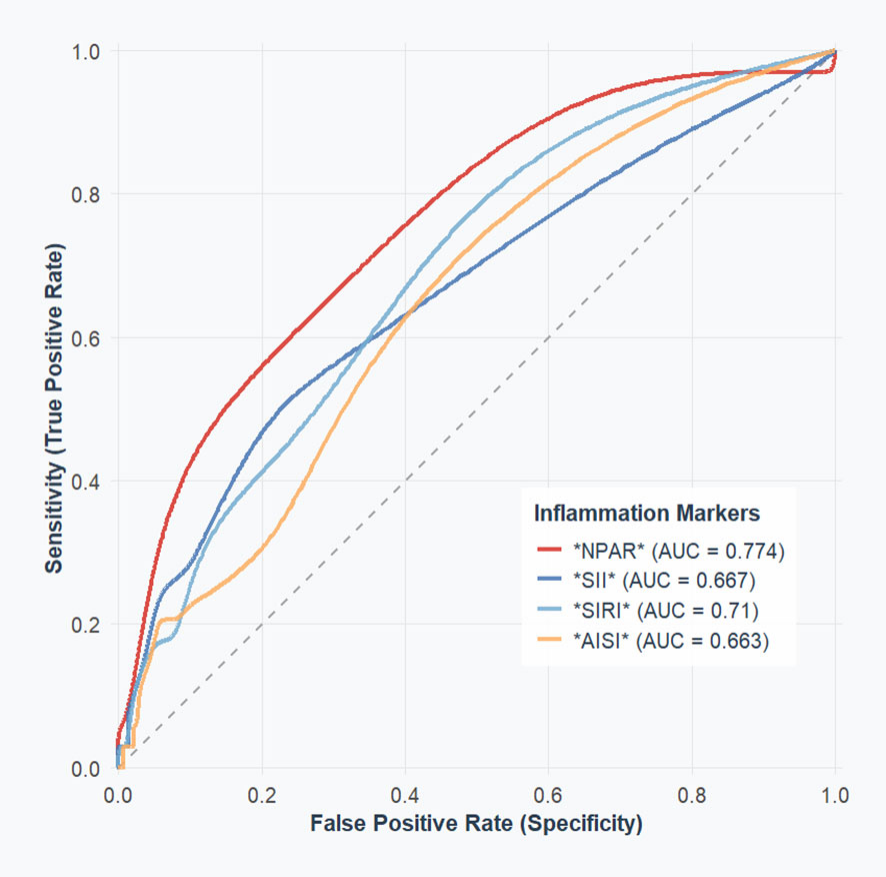
The ROC value of composite inflammatory ratios in predicting severity of the patient’s disease.

With an AUC of 0.774 (95% CI 0.6854 - 0.8620, *P* < 0.05), ROC analysis (**Figure 3**) revealed that the critical value of NPAR was 1.753, indicating a sensitivity of 0.848 and a specificity of 0.560 for diagnosing acute myocarditis. The critical value of SII for discriminating mild from severe cases was 1169, with a sensitivity of 0.783 and a specificity of 0.529, and an AUC of 0.667 (95% CI 0.5599 - 0.7749; *P* < 0.05). The cutoff value of SIRI was 2.058, with a sensitivity of 0.549 and a specificity of 0.852, and an AUC of 0.710 (95% CI 0.6201 - 0.8000, *P* < 0.05). The threshold value of AISI was 446, with a sensitivity of 0.570 and a specificity of 0.735, with an AUC of 0.663 (95% CI 0.5677 - 0.7587, *P* < 0.05).

To investigate if higher NPAR, SII, SIRI, and AISI were independent risk markers for patients with fulminant myocarditis, variables with *P* < 0.05 during the binary logarithmic analysis were included in the multivariate analysis (**Table 3**). High NPAR (OR = 5.67, 95%CI: 1.81 ~ 16.36, *P* < 0.05), SII (OR = 1.01, 95%CI: 1.01 ~ 1.01, *P* < 0.05), SIRI(OR = 1.21 95%CI: 1.06 ~ 1.37, *P* < 0.05), and AISI(OR = 1.01, 95%CI: 1.01 ~ 1.01, *P* < 0.05) were found to be independently linked with the probability of fulminant myocarditis in multivariate logistic regression analysis.

**Table 3:** Univariate and multivariate logistic analysis of complex inflammation ratio predicting fulminant myocarditis.

Variables	Model1		Model2		Model3	
	OR (95%CI)	*P*	OR (95%CI)	*P*	OR (95%CI)	*P*
NPAR	6.72 (2.82 ~ 16.01)	<.001	6.07 (2.32 ~ 15.92)	<.001	5.44 (1.81 ~ 16.36)	0.003
SII	1.01 (1.01 ~ 1.01)	0.002	1.01 (1.01 ~ 1.01)	0.008	1.01 (1.01 ~ 1.01)	0.010
SIRI	1.15 (1.04 ~ 1.26)	0.005	1.18 (1.07 ~ 1.30)	<.001	1.21 (1.06 ~ 1.37)	0.005
AISI	1.01 (1.01 ~ 1.01)	0.035	1.01 (1.01 ~ 1.01)	0.008	1.01 (1.01 ~ 1.01)	0.007

Model1: Crude.

Model2: Adjust: gender, age.

Model3: Adjust: Adjust: gender, age, smoking, drinking, hypertension, diabetes, coronary heart disease, EF%, cTnI.

## Discussion

Current diagnostic approaches for myocarditis remain nonspecific, primarily dependent on established clinical criteria rather than definitive biomarkers (13). Although the exact pathophysiological mechanisms remain incompletely understood, immune cells emerge as central orchestrators of myocardial inflammation. The inflammatory cascade in acute myocarditis involves coordinated actions of multiple immune lineages - neutrophils, monocytes, macrophages, and T/B lymphocytes (5, 16–18). Neutrophils, the most abundant circulating immune cells, serve as the first line of defense against pathogens and play a critical role in innate immune responses. Notably, their activity is amplified in acute myocarditis, exacerbating inflammatory cascades (6, 19). Experimental models demonstrate neutrophil chemotactic leadership, with these cells preceding monocyte infiltration and facilitating subsequent myeloid recruitment to inflamed myocardium (20). Treg cells play an important protective role in the occurrence of myocarditis (21). It was found that Treg cell abundance was negatively correlated with myocarditis severity (22, 23). Consistent with this paradigm, our cohort revealed marked lymphopenia in fulminant cases compared to moderate presentations, suggesting compromised immunoregulatory capacity. Oxidative stress plays an important role in the course of the disease in acute myocarditis, and serum albumin is the most abundant antioxidant in the whole blood (24). Albumin can combine lipopolysaccharides and other bacterial products (lipophosphomic acid and peptidoglycan), reactive oxygen species, nitric oxide and other nitrogen-reactive substances, and prostaglandins to regulate inflammation (25). Its levels fluctuate rapidly during acute inflammation due to extracellular metastasis (26). Therefore, hypoalbuminemia plays an important role in the development and progress of cardiovascular diseases (27). Our results also demonstrate that patients with fulminant myocarditis have lower serum albumin levels than those with mild myocarditis.

A meta-analysis by Ghulam et al. (28) identifies CRP as a dual-purpose biomarker for myocarditis diagnosis and outcome prediction. Emerging inflammatory indices combine practical advantages (cost-effectiveness, rapid assessment) with comprehensive immune-inflammation profiling, demonstrating independent prognostic value across multiple disease states (29–31). Recent researches find novel inflammatory biomarkers as significant predictors of both disease onset and adverse outcomes in cardiovascular conditions including CAD and hypertension (32–34). Cui et al. identified elevated NPAR at admission as an independent predictor of in-hospital mortality in ST-segment elevation myocardial infarction (STEMI) patients (35). Population-level research demonstrates significant associations between SII and SIRI with cardiovascular disease prevalence and all-cause mortality (36). Jiang et al. (12) revealed that elevated AISI levels in acute myocardial infarction (AMI) patients correlate with increased cardiovascular mortality risk, suggesting its utility as an early prognostic indicator. These findings collectively establish systemic inflammation biomarkers as critical tools for cardiovascular disease stratification and outcome prediction.

While NLR and MLR have established associations with myocarditis severity (8), the prognostic potential of novel composite inflammatory indices - NPAR, SII, SIRI, and AISI - remains underexplored. Nevertheless, research on compound inflammatory markers such NPAR, SII, SIRI, and AISI is lacking. This study provides the first analysis linking these advanced inflammatory indices to disease severity in acute myocarditis. Our findings reveal correlations between NPAR, SII, SIRI, and AISI levels with both cardiac dysfunction metrics and clinical severity stratification. Moreover, univariate and multivariate binary logistic regression results show that NPAR, SII, SIRI, and AISI are independently linked to patients’ risk of developing fulminant myocarditis. To our knowledge, this represents the inaugural investigation establishing NPAR, SII, SIRI, and AISI as clinically significant biomarkers for acute myocarditis severity assessment.

Our results indicate that elevated NPAR, SII, SIRI, and AISI levels at admission correlate with reduced cardiac function and heightened fulminant myocarditis risk. These indices may thus facilitate risk-stratified therapeutic strategies and serve as practical clinical tools for evaluating disease progression and potential complications.

### Strengths and limitations

There are several strengths and limitations in this study. To make sure the correlations are reliable and applicable to a wider range of people, we controlled for laboratory, examinational, and demographic factors. Secondly, even after controlling for a number of variables, residual or unmeasured confounding cannot be completely ruled out. Thirdly, our study did not consider treatment modalities, which may affect inflammatory indexes and the severity of myocarditis. Fourthly, the critical values obtained by ROC analysis for the diagnosis of explosive myocarditis were moderately sensitive and specific. Therefore, inflammation indicators can provide a certain reference for clinicians to evaluate the admission of acute myocarditis, but they still need to be comprehensively considered in combination with other indicators. Last but not least, this is a single-center retrospective analysis, rather than a multicenter clinical study, with a relatively small sample size. Therefore, prospective multicenter studies with a larger sample size are needed to validate these results.

## Conclusions

In conclusion, we are the first investigation to apply NPAR, SII, SIRI, and AISI as clinically significant biomarkers for acute myocarditis severity stratification. NPAR, SII, SIRI, and AISI indicators will provide theoretical support for early intervention in patients with acute myocarditis. These findings provide reference for the implementation of risk-adapted treatment options and early targeted interventions that promote acute myocarditis management.

## Data Availability

The original contributions presented in the study are included in the article/supplementary material. Further inquiries can be directed to the corresponding author/s.
